# The Influenza

**Published:** 1843-08

**Authors:** 


### THE INFLUENZA.

This affection, which has prevailed to the North and East, made its appearance in this city and neighborhood about the 1st of July. Out of the entire population of the city, we presume scarce a family escaped it. The milder cases were attended by slight soreness of the fauces, nasal passages, and chest, with occasional cough. In the graver cases, these symptoms were heightened, and accompanied by severer and general muscular or articular pains, great lassitude, and considerable arterial excitement. In a third variety, the mucous membranes generally seemed to be involved: in many instances there was great irritation of the alimentary canal, with mucous, serous, and sometimes bloody discharges. On the whole, however, considering the number attacked, we may say the epidemic was mild. For the most part, rest for a few days, and an occasional laxative, were entirely sufficient for the cure. A few cases required a more vigorous treatment—as detraction of blood and other antiphlogistic remedies.

The epidemic has now quite disappeared. We learn from the public prints that it is prevailing in St. Louis and New Orleans, and in all the intermediate towns. C.

